# Tramadol for chronic pain in adults: protocol for a systematic review with meta-analysis and trial sequential analysis of randomised clinical trials

**DOI:** 10.1186/s13643-023-02307-0

**Published:** 2023-08-22

**Authors:** J. Barakji, S. K. Korang, J. B. Feinberg, M. Maagaard, O. Mathiesen, C. Gluud, J. C. Jakobsen

**Affiliations:** 1 Copenhagen Trial Unit, Centre for Clinical Intervention Research, The Capital Region, Copenhagen University Hospital – Rigshospitalet, Blegdamsvej 9, 2100 Copenhagen Ø, Denmark; 2 Medical Department, Cardiology Section, Holbaek University Hospital, Holbaek, Denmark; 3 Department of Anaesthesiology, Centre for Anaesthesiological Research, Zealand University Hospital, Køge, Denmark; 4 Department of Clinical Medicine, Copenhagen University, Copenhagen, Denmark; 5 Department of Regional Health Research, The Faculty of Heath Sciences, University of Southern Denmark, Odense, Denmark

## Abstract

**Background:**

Chronic pain in adults is a frequent clinical symptom with a significant impact on patient well-being. Therefore, sufficient pain management is of utmost importance. While tramadol is a commonly used pain medication, the quality of evidence supporting its use has been questioned considering the observed adverse events. Our objective will be to assess the benefits and harms of tramadol compared with placebo or no intervention for chronic pain.

**Methods/design:**

We will conduct a systematic review of randomised clinical trials with meta-analysis and trial sequential analysis to assess the beneficial and harmful effects of tramadol in any dose, formulation, or duration. We will accept placebo or no intervention as control interventions. We will include adult participants with any type of chronic pain, including cancer-related pain. We will systematically search the Cochrane Library, MEDLINE, EMBASE, Science Citation Index, and BIOSIS for relevant literature. We will follow the recommendations by Cochrane and the Preferred Reporting Items for Systematic Review and Meta-Analysis (PRISMA) statement. The risk of systematic errors (‘bias’) and random errors (‘play of chance’) will be assessed. The certainty of evidence will be evaluated using the Grading of Recommendations Assessment, Development, and Evaluation (GRADE) approach.

**Discussion:**

Although tramadol is often being used to manage chronic pain conditions, the beneficial and harmful effects of this intervention are unknown. The present review will systematically assess the current evidence on the benefits and harms of tramadol versus placebo or no intervention to inform clinical practice and future research.

**Systematic review registration:**

PROSPERO CRD42019140334.

**Supplementary Information:**

The online version contains supplementary material available at 10.1186/s13643-023-02307-0.

## Background

### Description of pain

Pain may be defined as ‘an unpleasant sensory and emotional experience associated with actual or potential tissue damage or described in terms of such damage’ [1]. Pain is the most reported symptom in the general population and in medical settings [2–4]. Especially, persistent pain is a major international health problem [5], prompting the World Health Organization (WHO) to endorse a global campaign against pain [6]. Pain is the leading reason for patients using alternative medicines (e.g. acupuncture) [7]. Pain has been associated with a low degree of health-related quality of life and may lead to psychosocial distress, insomnia, and depressive symptoms [8–16]. Pain is also among the most common reasons for temporary or permanent work disability [17].

Pain may be caused by or be related to different clinical disorders and classified according to several different characteristics as well as according to the time span [18–21]. Below, we describe some of the most important classifications.

### Pain types defined according to duration and/or intensity of onset

#### Acute and chronic pain

Pain may be classified as ‘acute pain’ or ‘chronic pain’.

- Acute pain usually has a well-defined onset and most often a readily identifiable cause (e.g. surgery). Acute pain is expected to run its course in a short time frame, and management typically focuses on symptomatic relief [22]. Acute pain is a common symptom, affecting between 37 and 84% of hospitalised patients [23].
- Chronic pain is defined as pain lasting for more than 3 months by the International Association of the Study of Pain (IASP) [24]. Chronic pain is a frequent condition and is estimated to affect 20% of people worldwide [25–28] and accounting for 15 to 20% of physician visits [29, 30]. It may have a well-defined onset related to tissue injury (e.g. surgery) and be mediated through an intact nervous system. It may, however, also be caused by nerve damage and dynamic changes in the nervous system and be characterised by an ill-defined onset and a prolonged, fluctuating course [22]. When acute pain persists beyond the normal healing time, it may become chronic [31]. While persistent postoperative pain has a well-defined debut of pain, the transition to chronic pain is more indistinct [32]. A patient with chronic pain may not appear to be in pain, and the only definitive way to determine the presence of pain is to obtain a verbal report from the patient [22]. Clinically, pain is usually regarded as chronic when it lasts or recurs for more than 3 to 6 months [33, 34], but a recent systematic review demonstrated considerable heterogeneity in the criteria for a diagnosis of chronic pain applied in large epidemiological studies [35].

### Pain types defined according to condition

#### Cancer-related pain

Pain may also be classified based on whether it is cancer-related or not cancer-related. Cancer-related pain is caused by the cancer itself (primary tumour and metastases) or its treatment (e.g. radiation therapy) [36].

#### Postoperative pain

Postoperative pain is acute pain and includes direct nociceptive pain from tissue trauma, and pain from inflammation related to tissue trauma (i.e. surgical incision, dissection, burns) or direct nerve injury (i.e. nerve transection, stretching, or compression) [37]. Inflammation may also cause activation and sensitisation of pain pathways, resulting in primary and secondary hyperalgesia and central sensitisation, which is characterised by clinically increased pain, allodynia, and increased sensitivity from surrounding non-damaged anatomical areas [38].

#### Headache

Up to 90% of all patients with headaches may be classified as suffering from either tension-type headache, migraine, or cluster headache. While episodic tension-type headache is the most frequent headache type in population-based studies, migraine is the most common diagnosis in patients presenting to primary care physicians with headache [39].

#### Other types of pain

Pain in one or more anatomic regions where the aetiology is unknown is defined as idiopathic pain or primary pain [40]. Examples of idiopathic pain are chronic widespread pain, fibromyalgia, irritable bowel syndrome, and back pain that is not diagnosed as musculoskeletal or as neuropathic pain [24].

### Pain types defined according to specific mechanisms causing the pain

#### Somatic nociceptive pain

Nociceptive pain is the most frequent type of pain. It results from activity in neural pathways caused by actual tissue damage or potentially tissue-damaging stimuli [29, 41] originating from somatic nociceptors from skin, bone, joints, or muscles [42–45].

#### Visceral nociceptive pain

The visceral nociceptive pain is pain resulting from the viscera in the thoracic, pelvis, or abdominal organs. Visceral pain is diffuse, less distinctive, and difficult to localise. It is often characterised by referred visceral pain and followed by symptoms from the autonomic nerve system (e.g. nausea, sweating, cardiovascular symptoms) [46].

#### Neuropathic pain

The International Association for the Study of Pain defined neuropathic pain as ‘pain that arises as a direct consequence of a lesion or disease affecting the somatosensory system’ [47]. Neuropathic pain leads to a heterogeneous group of symptoms, including unremitting and spontaneous burning or shooting sensations, abnormal pain sensation to normal and harmless stimuli (allodynia), or a raised sensitivity to noxious stimuli (hyperalgesia) [48].

Neuropathic pain may be classified as central neuropathic pain or peripheral neuropathic pain. Central neuropathic pain conditions are mainly attributed to multiple sclerosis and post-stroke pain [49], while peripheral neuropathic pain is largely due to post-herpetic neuralgia and diabetic neuropathy [50]. Persistent postoperative pain (incidence up to 10% of surgical patients) may mostly be considered as iatrogenic neuropathic pain [32].

### Description of the intervention

Tramadol (tramadol hydrochloride) is a widely used opioid analgesic [51]. The total amount of tramadol used worldwide in the period from 1990 to 2009 was calculated to be 11,758 million defined daily doses (1 DDD defined as 300 mg) according to records of the manufacturers [52].

### Molecular mechanisms

Tramadol is an opioid agonist that also blocks the reuptake of serotonin and norepinephrine in the periphery [53, 54]. Tramadol exists as the racemic (1:1) mixture of the ( +) and ( −) enantiomer. Tramadol acts on the µ-opioid receptor via the *O*-desmethyl metabolite of tramadol (called M1 or ODT) and acts on the serotonin and noradrenaline reuptake via the ( +) and ( −) enantiomer [55–57]. Tramadol is metabolised in the liver by demethylation, oxidation, and conjugation [51, 53, 58]. Twenty-three metabolites have been identified [58], and both *O*- and *N*-desmethyl metabolites are formed. *O*-demethylation occurs primarily by the hepatic enzyme cytochrome P450 2D6 (CYP2D6) and *N*-demethylation by cytochrome P450 3A4 (CYP3A4) [59, 60]. Around 40% of the analgesic action is provided by *O*-desmethyl tramadol (M1) created by the rapid metabolism of tramadol in the liver via the cytochrome P450 enzyme CYP2D6 [53, 61, 62]. The CYP2D6 enzyme displays genetic polymorphism [51]. The prevalence of poor metabolisers in black populations has been estimated from 0 to 19%, compared to consistent reports of poor metaboliser status in Caucasians (5 to 10%) and Asians (0 to 2%) [63]. Other drugs metabolised by CYP2D6 enzymes (e.g. ondansetron) can potentially interfere with tramadol metabolism, changing the analgesic efficacy of tramadol [51].

### Clinical profile

Tramadol is associated with typical opioid adverse effects such as nausea, dizziness, and dry mouth, although vomiting and constipation are considered to be less of a problem as compared with traditional opioids [51, 64, 65]. Tramadol may hypothetically have a lower risk of dependence than conventional opioids but carries the risk of serotonin syndrome, especially when combined with other serotonergic agents [53, 55]. The risk of respiratory depression appears to be low compared with or to other opioids like morphine, pethidine, and oxycodone [52, 53, 64]. Tramadol is available in a variety of pharmaceutical formulations for oral (tablets, capsules), sublingual (drops), intranasal, rectal (suppositories), intravenous, subcutaneous, and intramuscular administration. The recommended maximum 24-h total oral daily dosage is up to 400 mg [66].

### Applicability of tramadol

Tramadol is used to treat moderate to severe pain [67, 68]. It has a wide range of applications in both acute (e.g. postoperative, trauma) and chronic (cancer and non-cancer) pain [52, 53, 60, 69–71] and is available in most countries worldwide [68].

Tramadol is listed in many medical guidelines for pain treatment [72]. It is mentioned as a step 2 analgesic in the WHO guidelines for cancer pain relief [73]. In chronic non-cancer pain, tramadol is recommended when non-opioid analgesics are ineffective or contraindicated [68].

### Adults

Due to the difference in pharmacodynamics and dosing of tramadol in children and adults, it does not seem reasonable to gather these trials together in a meta-analysis [74]. Because tramadol is a prodrug dependent of CYP2D6 activity, the risk of fatal overdosing seems considerably higher than with morphine [74]. Morphine is therefore generally considered as the 1st choice opioid treatment in children with acute pain in Denmark and worldwide.

### Why it is important to do this review

We have identified five previous reviews with meta-analyses assessing the effects of tramadol versus placebo on different types of pain [51, 75–78]. Three of these reviews assessed the effects of tramadol for neuropathic pain [51, 75, 77], two for nociceptive pain [76, 77], one for fibromyalgia-related pain [77], and one for cancer pain [78]. Two out of the six previous reviews used predefined Cochrane methodology [51, 78], and three used the GRADE approach for the assessment of certainty [51, 75, 78]. All the previous reviews only included randomised clinical trials, but none of these reviews systematically assessed the risks of bias of the included trials nor did they take into account the risks of random errors [51, 75–78]. None of the previous reviews used trial sequential analysis to assess imprecision.

In Additional file 1: Table S1, we have summarised the study design, results, and conclusions of the previous reviews. These previous reviews showed that common adverse events associated with tramadol are nausea, vomiting, dizziness, constipation, somnolence, and tiredness [51, 75–78]. Serious adverse events (e.g. abuse, death) were not reported consistently in the previous reviews.

The conclusions of the previous reviews differed as five of the reviews concluded that tramadol versus placebo was effective against pain [51, 75–77] and one of the reviews concluded that more evidence is needed to draw conclusions [78].

By combing randomised clinical trials across all types of chronic pain, we will increase the power and precision of the overall analysis and make it possible to conduct subgroup analyses comparing the effects of tramadol for different types of chronic pain. Sensitivity analyses may identify pain areas where tramadol could be especially beneficial and cause the least harm.

Our trial sequential analysis will assist in calculating the required information size thereby quantifying the statistical reliability of our data in the cumulative meta-analysis while adjusting the significance levels for sparse data.

### Objective

The objective of our review will be to assess the beneficial and harmful effects of tramadol versus placebo or no intervention for any type of chronic pain including cancer-related pain.

## Methods

This systematic review protocol has been developed based on the Preferred Reporting Items for Systematic Reviews and Meta-Analysis Protocols (PRISMA-P) guidelines for reporting systematic reviews evaluating healthcare interventions [79, 80]. A PRISMA-P checklist file is attached (Additional file 1).

### Criteria for considering studies for this review

#### Types of studies

We will include randomised clinical trials irrespective of trial design, setting, publication status, publication year, and language.

#### Types of participants

We will include adult participants with any type of chronic pain, i.e. chronic neuropathic pain, chronic nociceptive pain, chronic cancer-related pain, or any other types of chronic pain (as defined by the trialists). Participants will be included if > 18 years of age, irrespective of sex, and comorbidities.

#### Types of interventions

##### Experimental interventions

*Control interventions*: placebo or no intervention.

*Co-interventions*: we will accept any co-intervention but only if this co-intervention is delivered similarly in both groups.

#### Types of outcome measures

The following are the primary outcomes:

- Pain level assessed on visual analogue scale (VAS) or numerical rating scale (NRS).
- Proportion of participants with a serious adverse event defined as any untoward medical occurrence that resulted in death, was life-threatening, was persistent, or led to significant disability, nephrotoxicity, superinfection, need for respiratory support, need for circulatory support, or prolonged hospitalisation [81]. As we expect the trialists’ reporting of serious adverse events to be heterogeneous and not strictly according to the ICH-GCP recommendations, we will include the event as a serious adverse if the trialists either (1) use the term ‘serious adverse event’ but not refer to ICH-GCP or (2) report the proportion of participants with an event we consider fulfils the ICH-GCP definition (e.g. myocardial infarction or hospitalisation). If several of such events are reported, then we will choose the highest proportion reported in each trial.
- Quality of life measured on any valid continuous scale.

The following are the secondary outcomes:

- Dependence (as defined by trialists)
- Depressive symptoms (e.g. Hamilton Depression Rating Scale)
- Abuse (as defined by trialists)
- Proportion of participants with one or more adverse events not considered to be serious

We will for all outcomes use the trial results reported at maximal follow-up.

### Search methods for identification of studies

#### Electronic searches

We will search the Cochrane Central Register of Controlled Trials (CENTRAL), Medical Literature Analysis and Retrieval System Online (MEDLINE), Excerpta Medica Database (EMBASE), Latin American and Caribbean Health Sciences Literature (LILACS), Science Citation Index Expanded on Web of Science, and BIOSIS in order to identify relevant trials.

We will search all databases from their inception to the present.

#### Searching other resources

The reference lists of relevant publications will be checked for any unidentified randomised clinical trials. We will contact the authors of included trials and major pharmaceutical companies, by email asking for unpublished randomised clinical trials. Furthermore, we will search for ongoing trials on the following:

- ClinicalTrials.gov (www.clinicaltrials.gov)
- Google Scholar (https://scholar.google.dk/)
- The Turning Research into Practice (TRIP) Database (https://www.tripdatabase.com/)
- European Medicines Agency (EMA) (http:// www.ema.europa.eu/ema/)
- United States Food and Drug Administration (FDA) (www.fda.gov)
- China Food and Drug Administration (CFDA) (http://eng.sfda.gov.cn/WS03/CL0755/)
- Medicines and Healthcare Products Regulatory Agency (https://www.gov.uk/government/organisations/ medicines-and-healthcare-products-regulatory-agency)
- The World Health Organization (WHO) International Clinical Trials Registry Platform (ICTRP) search portal (http://apps.who.int/trialsearch/)

We will also consider relevant for the review of unpublished and grey literature trials if we identify these. To assess the magnitude of adverse events, we will attempt to uncover reports from regulatory authorities.

### Data collection and analysis

We will perform the review based on the recommendations of Cochrane [82]. The analyses will be performed using Review Manager 5 [83] and trial sequential analysis [84]. In case the Review Manager statistical software is not being sufficient, we will use STATA 16 [85].

### Selection of studies

Two authors (JB, SKK) will independently screen the titles and abstracts. We will retrieve all relevant full-text study reports/publications, and two review authors will independently screen the full text and identify and record reasons for the exclusion of the ineligible studies. We will resolve any disagreement through discussion, or if required, we will consult a third person (JCJ). Trial selection will be displayed in an adapted flow diagram as per the Preferred Reporting Items for Systematic Reviews and Meta-Analyses (PRISMA) statement [86].

### Data extraction and management

Four authors (JB, SKK, JBF, MM) will in pairs extract data independently from included randomised clinical trials. Disagreements will be resolved by discussion with a fifth author (JCJ). We will assess duplicate publications and companion papers of a trial together to evaluate all available data simultaneously (maximise data extraction, correct bias assessment). We will contact the trial authors by email to specify any additional data, which may not have been reported sufficiently or at all in the publication.

### Trial characteristics

The following are the trial characteristics: bias risk components (as defined below), trial design (parallel, factorial, or crossover), number of intervention arms, length of follow-up, estimation of sample size, inclusion criteria, exclusion criteria, study design (placebo or no intervention), and trial duration and follow‐up.

### Participant characteristics and diagnosis

The following are the participant characteristics and diagnosis: number of randomised participants, number of analysed participants, number of participants lost to follow-up/withdrawals/crossover, compliance with medication, age range (mean or median) and sex ratio, type of chronic pain, baseline pain score, drug and dosing regimen, study design (placebo or no intervention) trial duration and follow‐up, analgesic outcome measures and results, proportion of participants with one or more serious adverse events, and proportion of participants with one or more non-serious adverse events.

### Co-intervention characteristics

The following are the co-intervention characteristics: type of co-intervention, dose of co-intervention, duration of co-intervention, and mode of administration.

### Notes

Funding of the trial and notable conflicts of interest of trial authors will be extracted, if available.

We will note in the ‘Characteristics of included trials’ table if outcome data were not reported in a usable way. Four review authors will independently transfer data into the Review Manager file [83]. Disagreements will be resolved through discussion, or if required, we will consult with a fifth author.

### Assessment of risk of bias in included studies

We will use the instructions given in the Cochrane Handbook for Systematic Reviews of Interventions [82] in our evaluation of the methodology and hence the risk of bias of the included trials. We will evaluate the methodology in respect of the following:

- Random sequence generation
- Allocation concealment
- Blinding of participants and personnel
- Blinding of outcome assessor
- Incomplete outcome data
- Selective outcome reporting
- Overall risk of bias

These domains enable the classification of randomised trials at low risk of bias and at high risk of bias. The latter trials tend to overestimate positive intervention effects and underestimate negative effects [87–93].

We will classify the trials according to the following criteria.

Random sequence generation:

- Low risk: If sequence generation was achieved using a computer random number generator or a random number table. Drawing lots, tossing a coin, shuffling cards, and throwing dice were also considered adequate if performed by an independent adjudicator.
- Unclear risk: If the method of randomisation was not specified, but the trial was still presented as being randomised.
- High risk: If the allocation sequence is not randomised or only quasi-randomised. These trials will be excluded.

Allocation concealment:

- Low risk: If the allocation of patients was performed by a central independent unit, on-site locked computer, identical-looking numbered sealed envelopes, drug bottles, or containers prepared by an independent pharmacist or investigator.
- Uncertain risk: If the trial was classified as randomised but the allocation concealment process was not described.
- High risk: If the allocation sequence was familiar to the investigators who assigned participants.

Blinding of participants and personnel:

- Low risk: If the participants and the treatment providers were blinded to the intervention allocation and this was described.
- Uncertain risk: If the procedure of blinding was insufficiently described.
- High risk: If blinding of participants and the treatment providers was not performed.

Blinding of outcome assessors:

- Low risk of bias: If it was mentioned that outcome assessors were blinded, and this was described.
- Uncertain risk of bias: If it was not mentioned if the outcome assessors in the trial were blinded or the extent of blinding was insufficiently described.
- High risk of bias: If no blinding or incomplete blinding of outcome assessors was performed.

Incomplete outcome data:

- Low risk of bias: If missing data were unlikely to make treatment effects depart from plausible values. This could be either (1) there were no drop-outs or withdrawals for all outcomes or (2) the numbers and reasons for the withdrawals and drop-outs for all outcomes were clearly stated and could be described as being similar to both groups. Generally, the trial is judged as at a low risk of bias due to incomplete outcome data if drop-outs are less than 5%. However, the 5% cut-off is not definitive.
- Uncertain risk of bias: If there was insufficient information to assess whether missing data were likely to induce bias on the results.
- High risk of bias: If the results were likely to be biassed due to missing data either because the pattern of drop-outs could be described as being different in the two intervention groups or the trial used improper methods in dealing with the missing data (e.g. last observation carried forward).

Selective outcome reporting:

- Low risk of bias: If a protocol was published before or at the time the trial was begun and the outcomes specified in the protocol were reported on. If there is no protocol or the protocol was published after the trial has begun, reporting a clinically important pain-relieving effect and serious adverse events will grant the trial a grade of low risk of bias.
- Uncertain risk of bias: If no protocol was published and the outcome pain assessment on VAS or NRS and serious adverse events were not reported.
- High risk of bias: If the outcomes in the protocol were not reported.

Overall risk of bias:

- Low risk of bias: The trial will be classified as overall ‘low risk of bias’ only if all of the bias domains described in the above paragraphs are classified as ‘low risk of bias’.
- High risk of bias: The trial will be classified as ‘high risk of bias’ if any of the bias risk domains described in the above are classified as ‘unclear’ or ‘high risk of bias’.

We will assess the domains ‘Blinding of outcome assessment’, ‘Incomplete outcome data’, and ‘Selective outcome reporting’ for each outcome result. Thus, we can assess the bias risk for each outcome assessed in addition to each trial. Our primary conclusions will be based on the results of our primary outcome results with an overall low risk of bias. Both our primary and secondary conclusions will be presented in the summary of findings tables.

### Differences between the protocol and the review

We will conduct the review according to this published protocol and report any deviations from it in the ‘Differences between the protocol and the review’ section of the systematic review.

### Measures of treatment effect

#### Dichotomous outcomes

We will report risk ratios (RRs) with a 97.5% confidence interval (CI) for primary dichotomous outcomes, as well as trial sequential analysis-adjusted CIs (see ‘meta-analysis for details’).

#### Continuous outcomes

We will report mean differences (MDs) with 97.5% CI for primary continuous outcomes and consider reporting standardised mean differences (SMDs) with 95% CI for continuous outcomes. We will also report trial sequential analysis-adjusted CIs (see below).

### Dealing with missing data

We will, as the first option, contact all trial authors to obtain any relevant missing data (i.e. for data extraction and for assessment of risk of bias, as specified above).

#### Dichotomous outcomes

We will not impute missing values for any outcomes in our primary analysis. In our sensitivity analyses (see paragraph below), we will impute data on serious adverse events.

#### Continuous outcomes

We will primarily analyse scores assessed at single time points. If only change from baseline scores are reported, we will analyse the results together with follow-up scores [82]. If standard deviations (SDs) are not reported, we will calculate the SDs using trial data, if possible. We will not use intention-to-treat data if the original report did not contain such data. We will not impute missing values for any outcomes in our primary analysis. In our sensitivity analysis (see paragraph below) for continuous outcomes, we will impute data on group mean difference and standard deviation.

### Assessment of heterogeneity

We will primarily investigate forest plots to visually assess heterogeneity. We will secondly assess the presence of statistical heterogeneity by the chi^2^ test (threshold *p* < 0.05) and measure the quantities of heterogeneity by the *I*^2^ statistic and tau (*τ*)^2^ statistic [94, 95].

We will investigate the reasons for heterogeneity through subgroup analyses. Ultimately, we may decide that a meta-analysis should be avoided [82].

### Assessment of reporting biases

We will use a funnel plot to assess reporting bias if ten or more trials are included. We will visually inspect funnel plots to assess the risk of bias. From this information, we assess the possible reporting bias. For dichotomous outcomes, we will test asymmetry with the Harbord test [96] if *τ*^2^ is less than 0.1 and with the Rücker test if *τ*^2^ is more than 0.1. For continuous outcomes, we will use the regression asymmetry test [97] and the adjusted rank correlation [98].

### Unit of analysis issues

We will only include randomised clinical trials. For trials using a crossover design, only data from the first period will be included [82, 99]. There will therefore not be any unit of analysis issues. We will not include cluster randomised trials.

### Minimal important difference

In clinical intervention research, it is of utmost importance always to define minimal important differences (MID) and to define thresholds for clinical significance [100]. If a large number of trial participants are analysed, small and clinically irrelevant intervention effects may lead to statistically significant results and rejection of the null hypothesis [101]. Jaeschke et al. defined the minimal important difference as ‘the smallest difference in score in the domain of interest which patients perceive as beneficial’ [102].

Estimations of minimal important differences should be used as arbitrary strict precise thresholds. However, to avoid erroneous conclusions, minimal important differences need to be estimated and predefined when assessing the effects of interventions for pain. Olsen et al. have conducted two systematic reviews on this matter in order to gather the evidence and present an estimate of the minimal important difference [103, 104]. Olsen et al. conducted a systematic review on the minimal important difference in patients with acute pain and concluded that the median of the studies’ results was 17 mm on VAS (IQR 14 to 23 mm) [103]. Another systematic review conducted by Olsen et al. was on the minimal important difference in patients with chronic pain, and the results showed a median of 23 mm on VAS (IQR 12 to 39 mm) when using the within-patient anchor-based method, while the median in studies using the sensitivity- and specificity-based method was 20 mm on VAS (IQR 15–30 mm) [104]. We have described detailed considerations about minimal important differences in Additional file 1: Appendix.

There is currently no agreement on an appropriate minimal important difference threshold in chronic pain nor the most suitable method to approximate this threshold [104].

In this systematic review, we will choose a minimal important difference equivalent of 10 mm on the visual analogue scale or 1 point and the numerical rating scale. Thereby, we are in close agreement with the lower interquartile range boundary of 12 mm or 1.2 cm of previous findings [104], in agreement with other choices with minimal important differences in other systematic reviews of chronic pain [105, 106] and avoid missing a clinically important effect.

### Data synthesis

#### Meta-analysis

We will undertake this meta-analysis according to the recommendations stated in the Cochrane Handbook for Systematic Reviews of Interventions [82], Keus et al. [107], and the eight-step assessment suggested by Jakobsen et al. [100]. We will use the statistical software Review Manager 5.3 [83] provided by Cochrane to analyse data. We will assess all our intervention effects with both random-effects meta-analyses [108] and fixed-effect meta-analyses [109]. We will primarily report the more conservative results (highest *p* value) of the two [100]. The least conservative results will be considered a sensitivity analysis. We will use three primary and four secondary outcomes, and therefore, we will consider a *p* value of 0.025 as the threshold for statistical significance [100, 110]. The corresponding confidence interval is 97.5%. We will investigate for heterogeneity through subgroup analyses. Ultimately, we may decide that a meta-analysis should be avoided [82]. We will use the eight-step procedure to assess if the thresholds for statistical and clinical significance are crossed [100]. Our primary conclusion will be based on the results at a low risk of bias [100].

Where multiple trial arms are reported in a single trial, we will include only the relevant arms. If two comparisons are combined in the same meta-analysis, we will halve the control group to avoid double-counting [82]. Trials with a factorial design will be included.

If quantitative synthesis is not appropriate, we will report the results in a narrative way.

#### Trial sequential analysis

Traditional meta-analysis runs the risk of random errors due to sparse data and repetitive testing of accumulating data when updating reviews. We wish to control the risks of type I errors and type II errors. We will therefore perform trial sequential analysis on the outcomes, in order to calculate the required information size (that is the number of participants needed in a meta-analysis to detect or reject a certain intervention effect) and the cumulative *Z*-curve’s breach of relevant trial sequential monitoring boundaries [84, 111–119]. A more detailed description of trial sequential analysis can be found in the trial sequential analysis manual [112] and at http://www.ctu.dk/tsa/. For dichotomous outcomes, we will estimate the required information size based on the observed proportion of patients with an outcome in the control group (the cumulative proportion of patients with an event in the control groups relative to all patients in the control groups), a relative risk reduction of 25%, an alpha of 2.5% for our primary and secondary outcomes, a beta of 10%, and diversity as suggested by the trials in the meta-analysis. For the outcome ‘pain level assessed on the visual analogue scale (VAS) or numerical rating scale (NRS)’, we will use a minimal important difference estimate based on previously conducted systematic reviews [103, 104]. We will accept a pain-relieving effect equivalent to 10 mm or 1 point on the visual analogue scale and the numerical rating scale, respectively.

For all remaining continuous outcomes, we will in the trial sequential analysis use the observed SD, a mean difference of the observed SD/2, an alpha of 2.5% for our primary and secondary outcomes, and a beta of 10%.

### Subgroup analyses and investigation of heterogeneity

#### Subgroup analyses

We will perform the following subgroup analysis when analysing the primary outcomes (pain level assessed on visual analogue scale (VAS) or numerical rating scale (NRS), serious adverse event, and quality of life).

1. Trials at a high risk of bias compared to trials at a low risk of bias
2. Trials at risk of vested interests compared to trials at no risk of vested interests
3. Trials compared according to the type of chronic pain
4. Trials compared according to dosage of tramadol used (below median compared to median and above)
5. Trials compared according to the duration of tramadol administration (below median compared to median and above)
6. Age of participants: 18 to 59 years compared to 60 to 79 years compared to above 80 years

We will use the formal test for subgroup differences in Review Manager [83].

#### Sensitivity analyses

To assess the potential impact of the missing data for dichotomous outcomes, we will perform the two following sensitivity analyses on our primary outcomes.

- ‘Best–worst-case’ scenario: We will assume that all participants who lost to follow-up in the tramadol intervention group have had no serious adverse event and that all those participants who lost to follow-up in the placebo group have had a serious adverse event.
- ‘Worst-best-case’ scenario: We will assume that all participants who lost to follow-up in the tramadol intervention group have had a serious adverse event and that all those participants who lost to follow-up in the placebo group have had no serious adverse event.

We will present the results of both scenarios in our review.

For all continuous outcomes when analysing a ‘beneficial outcome’ will be the group mean plus two standard deviations (SDs) (we will secondly use one SD in another sensitivity analysis) of the group mean and a ‘harmful outcome’ will be the group mean minus two SDs (we will secondly use one SD in another sensitivity analysis) of the group mean [100].

To assess the potential impact of missing SDs for continuous outcomes, we will perform the following sensitivity analysis.

- Where SDs are missing and it is not possible to calculate them, we will impute SDs from trials with similar populations and a low risk of bias. If we find no such trials, we will impute SDs from trials with a similar population. As the final option, we will impute SDs from all trials.

We will present the results of this scenario in our review. Other post hoc sensitivity analyses might be warranted if unexpected clinical or statistical heterogeneity is identified during the analysis of the review results [100].

### Summary of findings

We will create a Summary of Findings table using each of the primary outcomes (pain level assessed on VAS or NRS, serious adverse event, and quality of life). We will use the five GRADE considerations (bias risk of the trials, consistency of effect, imprecision, indirectness, and publication bias) to assess the certainty of the evidence as it relates to the studies which contribute data to the meta-analyses for the prespecified outcomes [100, 120–122]. We will assess imprecision by trial sequential analysis but will otherwise use methods and recommendations described in chapter 8 (Sect. 8.5) and chapter 12 of the Cochrane Handbook for Systematic Reviews of Interventions [82] using the GRADEpro software. We will downgrade imprecision in GRADE by two levels if the accrued number of participants is below 50% of the diversity-adjusted required information size (DARIS) and one level if between 50 and 100% of DARIS [123]. We will not downgrade if the cumulative *Z*-curve crosses the monitoring boundaries for benefit, harm, or futility, or DARIS is reached [123]. We will justify all decisions to downgrade the quality of studies using footnotes, and we will make comments to aid the reader’s understanding of the review where necessary. Firstly, we will present our results in the Summary of Findings table based on the results from the trials with a low risk of bias, and secondly, we will present the results based on all trials.

## Discussion

This protocol aims at investigating the beneficial and harmful effects of tramadol versus placebo or no intervention in adults with any type of chronic pain condition. The outcomes will be pain level assessed on VAS or NRS, serious adverse events, quality of life, dependence, depressive symptoms, abuse, and non-serious adverse events.

This protocol has several strengths. The predefined methodology is based on the Cochrane Handbook for Systematic Reviews of Interventions [82], the eight-step assessment suggested by Jakobsen et al. [100], trial sequential analysis [84], and GRADE assessment [120–122]. Hence, we will consider both the risks of random errors and the risks of systematic errors. We predefine evidence-based estimations of minimal important differences which will limit the risk of focusing on statistically significant results with questionable clinical importance. This threshold of minimal important difference is based on the estimations of several previously conducted studies and reviews [103, 104]. Compared to previous systematic reviews on tramadol, we want to assess the effects versus placebo for all different forms of chronic pain including cancer-related pain. This increases the power and precision of our analyses and makes it possible to conduct subgroup analyses and sensitivity analyses that may identify pain areas where tramadol could be especially beneficial and cause the least harm.

Our protocol also has several limitations. We include participants with all types of chronic pain including cancer-related pain; tramadol might have different effects on different types of chronic pain. Indeed, it might be problematic to combine trials assessing the effects of tramadol on chronic pain including cancer-related pain because of different underlying pathophysiological mechanisms [124]. On the other hand, the effects of tramadol on chronic pain might be comparable, and therefore, it might be valid to combine trials assessing the effects of tramadol on different types of chronic pain in meta-analysis, which would also increase the statistical power. Another potential limitation is that we only intend to include randomised clinical trials. The results of non-randomised studies are prone to show biassed results primarily due to confounding by indication and that is why we do not include non-randomised studies. However, rare and late-occurring adverse events are often not possible to identify in randomised trials. If tramadol shows areas of benefit then we plan to perform a systematic review assessing adverse events in observational studies [125].

Another limitation is that we are not going to assess randomised clinical trials evaluating tramadol versus other pain-reducing interventions, e.g. other morphine products and schedules or other ‘pain killers’. These jobs must be conducted once we have established the benefits of tramadol versus placebo or no intervention.

A further limitation is the large number of comparisons which increase the risk of type I errors. We have adjusted our thresholds for statistical significance according to the number of primary outcomes, but we have also included multiple subgroup analyses. The increased risk of type I errors will be considered when interpreting the review results.

### Supplementary Information

**Additional file 1. **Appendix.

## Data Availability

Not applicable.
