# Supplementary material for: Tramadol for chronic pain in adults: protocol for a systematic review with meta-analysis and trial sequential analysis of randomised clinical trials
Source: Syst Rev. 2023 Aug 22;12:145. doi: 10.1186/s13643-023-02307-0 (PMC10463795; doi:10.1186/s13643-023-02307-0)
Supplement: Supplementary file 1 — Additional file 1. Appendix. [file 13643_2023_2307_MOESM1_ESM.docx]

**Appendix**

**Tramadol for chronic pain in adults. Protocol for a systematic review with meta-analysis and Trial Sequential Analysis of randomised clinical trials**

Barakji J^1,*^, Korang SK^1^, Feinberg JB^1,2^ , Maagaard M^1,3^, Mathiesen O^3,4^, Gluud C^1,5^, Jakobsen JC^1,5^

^1^ Copenhagen Trial Unit, Centre for Clinical Intervention Research, The Capital Region, Copenhagen University Hospital ─ Rigshospitalet, Copenhagen, Denmark

^2^ Medical Department, Cardiology Section, Holbaek University Hospital, Holbaek, Denmark.

^3^ Centre for Anaesthesiological Research, Department of Anaesthesiology, Zealand University Hospital, Køge, Denmark

^4^ Department of Clinical Medicine, Copenhagen University, Copenhagen, Denmark

^5^ Department of Regional Health Research, The Faculty of Heath Sciences, University of Southern Denmark, Odense, Denmark

^*^Corresponding author

**Jehad Ahmad Barakji**

**Phone:** +45 21 52 07 80

**E-mail**: [jehad.barakji@ctu.dk](mailto:jehad.barakji@ctu.dk)

**Address:** Copenhagen Trial Unit, Centre for Clinical Intervention Research, The Capital Region, Copenhagen University Hospital ─ Rigshospitalet, Blegdamsvej 9, DK-2100 Copenhagen Ø, Denmark

**Competing interest** None known.

**Minimal important difference**

For the determination of minimal important differences in clinical trials two types of methods are available; anchor-based methods and distribution-based methods [1].

*Anchor-based methods*

Anchor-based methods relate the change in a person reported outcome score (e.g. a score on the visual analog scale (VAS)) to a subjective global assessment rating (e.g. scores from the Clinical Global Impressions-Improvement (CGI-I)) which is used as an ‘anchor’ [1]. Ideally, there needs to be an established association between the person reported outcome score and the ‘anchor’ to make any meaningful inference about a minimal important difference [2].

There are two subtypes of anchor-based methods, i.e., the ‘*within-patient score’* and the *‘between-patients score’* [1].

- Within-patient score defines minimal important difference as the average minimal change in a given person’s reported outcome score that leads to a clinically observable change in the subjective global assessment rating (the latter is used as an anchor) [1]. For example, to ascertain the minimal important difference regarding depression management, Moncrieff et al. describes the linking of within-patient scores (change from baseline) scores on the Hamilton Depression Rating Scale (the most commonly used depression rating scale) to scores on the Clinical Global Impressions-Improvement (CGI-I) scale, a scale which rates improvement on a scale of 1 (very much improved from baseline) through 4 (no change from baseline) to 7 (very much worse from baseline) [3]. Moncrieff et al. conclude that seven points on the Hamilton Depression Rating Scale (range typically from 0 to 54 points) correspond to a minimal important difference when using within-patient scores [3].
- The between-patients score method, also known as ‘the group difference’ method, compare the reported outcome scores between a group of people with no clinically observable change (based on a subjective global assessment rating (used as an anchor)) to a group of people with clinically observable change (based on a subjective global assessment rating (used as an anchor)). The minimal important difference is then estimated as the mean difference between these two groups [4]. For example, Musoro et al. defines the minimal important difference (MID) as the group difference in terms of quality of life assessed by HRQOL scores [5]. Participants were assigned to distinct subgroups reflecting various levels of change (e.g. no change, small positive changes, large positive changes, small negative changes, or large negative changes). The group difference was identified by the comparison of the average of the HRQOL scores of the group of participants with at ‘small change’ to the HRQOL scores of the group of participants with ‘no change’ [5].

There are also other anchor-based methods (e.g. the sensitivity- and specificity-based method and the social comparison method) [1]. The sensitivity- and specificity-based method aims to identify the minimal important difference that allows for the best discrimination between groups of patients (i.e. the score that produces the greatest sensitivity and specificity) [1]. For example, an outcome measure (e.g. Numerical rating scale score) is considered a ‘diagnostic test’ and the anchor (e.g. Global Perceived Effect) is used as gold standard. Hence, standard methods may be used to estimate sensitivity and specificity [1]. Sensitivity is the proportion of patients who report an improvement on the external criterion (the anchor) and these patients reported outcome scores above the threshold minimal important difference value [1]. Specificity is the proportion of patients who do not report an improvement on the external criterion (the anchor) and these patients reported outcome scores below the threshold minimal important difference value [1]. Receiver operating characteristic (ROC) curves are then used to identify the patients’ reported outcome score with the greatest sensitivity and specificity [6-8].

*The distribution-based methods*

Distribution-based methods are based on the statistical characteristics of the outcome in the obtained sample [9]. Crosby et al. [9] have identified two general types of distribution-based methods for estimations of minimal important differences:

- The first type of distribution-based method evaluate change in relation to sample variation [9]. Different types of variation can be used: effect size, standardised response mean, and responsiveness statistic [9]. The effect size represents individual change in relation to the number of pre-test standard deviations (SDs) [9]. Cohen et al. has suggested benchmarks to better interpret the effect sizes: 0.20 of SD for ‘small’ effects, 0.50 for ’moderate’ effects, and 0.80 for ’large’ effects [10]. Whereas the effect size is the ratio of individual change to the baseline standard deviation of the sample, standardised response mean is the ratio of individual change to the standard deviation of that change [11]. A large standardised response mean indicates that the change is large in comparison to the background variability in the measurements [9]. Guyatt et al. has proposed a responsiveness statistic as a variation of standardised response mean; calculated by dividing the difference between pre-test and post-test by the standard deviation of change observed for a group of stable participants [12].
- The second type distribution-based method is based on the measurement precision of the instrument [9]. This method include the standard error of the mean (SEM) and evaluate the change in relation to variation of the instrument as opposed to variation in the sample [9]. SEM is a measure of the precision of a test instrument and considered an attribute of the measure and not a characteristic of the sample per se [13]. SEM for a given measure is likely to vary across samples depending upon the method used to estimate reliability and the presence of extreme scores [9]. Different thresholds for a minimal important difference have been suggested, i.e. values of 1 SEM [14], 1.96 SEM [15], and 2.77 SEM [13, 15].

In conclusion, different methods for estimating minimal important differences exist, but no single method has been shown to be the optimal method. The question of whether to use anchor-based or distribution-based methods for determining clinically meaningful change has received considerable attention and debate [9]. Dworkin et al. defined the clinical importance of patient improvement as the clinically important changes in individuals that can be identified using either within-patient anchor-based method or distribution-based method [16, 17], while the clinical importance of group differences could be the clinical difference between a treatment group and a placebo group or between two different treatment groups [18]. Dworkin et al. claim that the clinical important difference identified in individuals cannot be directly extrapolated to the evaluation of group differences [17, 19-22]. The U.S. Food and Drug Administration also states in their web site “When defining meaningful change on an individual patient basis, that definition is generally larger than the minimum important difference for application to group mean comparisons” [22].

While it is claimed that the within-patient differences are larger than the between-group difference [22], based on the studies included in our review we are not able to find a significant difference between the minimal important difference estimated by the two different methods.

**Previously conducted reviews on this subject**

- Cepeda et al. concluded that the evidence concerning the analgesic effect of tramadol for osteoarthritic pain is strong, but the effects size is only modest [23]. Cepeda et al. also concluded that the usefulness of tramadol might be limited by adverse events [23]. Cepeda et al. did not publish a protocol before their review [23].
- Duehmke et al. and Finnerup et al. both concluded that tramadol might have a beneficial analgesic effect on neuropathic, but both reviews stated that the recommendation of tramadol is ‘weak’ because of poor quality evidence and high risks of adverse events [24, 25].
- Furlan et al. concluded that tramadol reduce pain intensity and improve functional outcomes in patients with fibromyalgia [26]. Furlan et al. also concluded that tramadol did not significantly outperform NSAIDs or tricyclic antidepressants for either pain relief or functional outcomes [26]. Opioids (including tramadol) in general were more effective than placebo for both nociceptive and neuropathic pain syndromes [26]. Furlan et al. did not publish a protocol on before their review [26].
- Wiffen et al. concluded that the use of tramadol for cancer pain is unclear due to lack of good quality trials [27].

| First author | Titel | Year of publication | Design | Types of participants | Information sources | No. of trials | No. of participants | Published protocol | Outcomes | Assessment of adverse events | Assessment of risk of bias | Accounts for random error | Use of the Grade Approach | Conclusion |
| --- | --- | --- | --- | --- | --- | --- | --- | --- | --- | --- | --- | --- | --- | --- |
| Finnerup et al. [24] | Pharmacotherapy for neuropathic pain in adults: a systematic review and meta-analysis | 2015 | Systematic review and meta-analysis | Neuropathic pain | PubMed, MEDLINE, Cochrane Library, EMBASE, FDA website EMEA website, and Clinicaltrials.gov | 229 with 7 trials using tramadol as the intervention | 16.721 with 741 tramadol patients | Unpublished, but available via Web address | Number needed to treat (NNT) for 50% pain intensity reduction (or 30% pain reduction or at least moderate pain relief) was the primary effect measure. Difference in pain intensity was a secondary outcome. | Yes | Yes | No | Yes | The findings permitted a weak recommendation for use and proposal as second line treatment in neuropathic pain for tramadol |
| Duehmke et al. [25] | Tramadol for neuropathic pain in adults | 2017 | Cochrane review | Neuropathic pain | Cochrane Library, MEDLINE, and EMBASE | 6 | 438 | Yes | Primary outcomes:  Participant-reported pain relief of 30% or greater.  Participant-reported pain relief of 50% or greater.  PGIC much or very much improved.  PGIC very much improved. | Yes | Yes, except for financial | No | Yes | The evidence of benefit from tramadol was of low or very low quality, meaning that it does not provide a reliable indication of the likely effect. |
| Cepeda et al. [23] | Tramadol for Osteoarthritis: A Systematic Review and meta-analysis | 2007 | Meta-analysis | Osteoathritis (nociceptive pain) | Cochrane Library, MEDLINE, EMBASE, and LILACS | 11 trials with 9 trials on tramadol alone. Intervention is compared with placebo, morphine, nalbuphine, pethidine, or fentanyl | 1939 | No | Effectiveness of oral tramadol for relieving pain and improving physical function in people with OA, second to assess the duration of any benefit, and third to determine the safety of tramadol. | Yes | Yes | No | No | Tramadol or tramadol/paracetamol decreases pain intensity, produces symptom relief, and improves function in patients with OA, but these benefits are small. |
| Furlan et al. [26] | Opioids for chronic non-cancer pain: a meta-analysis of effectiveness and side effects | 2006 | Meta-analysis | Nociceptive pain (osteoarthritis, rheumatoid arthritis or back pain), neuropathic pain (postherpetic neuralgia, diabetic neuropathy or phantom limb pain), and fibromyalgia related pain | MEDLINE, EMBASE, Cochrane Library, the ACP Journal Club, and DARE | 41 trials (with 17 of the trials on tramadol). Intervention is compared with placebo, clomipramine, levomepromazine, and diclofenac | 6019 (3433) | Not available | The data extracted were those quantifying pain (intensity or relief), function and side effects. | Yes | Yes, except publication bias | No | No | Weak (incl. tramadol) and strong opioids outperformed placebo for pain and function in all types of CNCP. Other drugs (i.e naproxen and nortriptyline) produced better functional outcomes than opioids, whereas for pain relief they were outperformed only by strong opioids. Despite the relative shortness of the trials, more than one-third of the participants abandoned treatment. |
| Wiffen et al. [27] | Tramadol with or without paracetamol (acetaminophen) for cancer pain | 2017 | Cochrane review | Cancer pain | Cochrane Library, MEDLINE, EMBASE, and LILACS | 10 trials (with 1 trial comparing tramadol with placebo) | 958 (119) | Yes | Primary outcomes:  Number of participants with pain reduction of 30% or greater from baseline.  Number of participants with pain reduction of 50% or greater from baseline.  Number of participants with pain no worse than mild  Number of participants with PGIC of much improved or very much improved (or equivalent wording). | Yes | Yes, except for financial bias | No | Yes | There is limited, very low quality, evidence from randomised controlled trials that tramadol produced pain relief in some adults with pain due to cancer and no evidence at all for children. The place of tramadol in managing cancer pain and its role as step 2 of the WHO analgesic ladder is unclear. |

**References**

1. Copay AG, Subach BR, Glassman SD, et al. Understanding the minimum clinically important difference: a review of concepts and methods*.* *Spine J*, 2007. **7**(5): p. 541-6.

2. Guyatt GH, Osoba D, Wu AW, et al. Methods to Explain the Clinical Significance of Health Status Measures*.* *Mayo Clinic Proceedings,* 2002. **77**(4): p. 371-383.

3. Moncrieff J and Kirsch I. Empirically derived criteria cast doubt on the clinical significance of antidepressant-placebo differences*.* *Contemp Clin Trials*, 2015. **43**: p. 60-2.

4. Hagg O, Fritzell P, and Nordwall A. The clinical importance of changes in outcome scores after treatment for chronic low back pain*.* *Eur Spine J*, 2003. **12**(1): p. 12-20.

5. Musoro ZJ, Hamel J, Ediebah DE,, et al. Establishing anchor-based minimally important differences (MID) with the EORTC quality-of-life measures: a meta-analysis protocol*.* *BMJ Open, 2018*. **8**(1).

6. Stratford PW, Binkley JM, Riddle DL, et al. Sensitivity to change of the Roland-Morris Back Pain Questionnaire: part 1*.* *Phys Ther*, 1998. **78**(11): p. 1186-96.

7. Riddle DL, Stratford PW, and Binkley JM. Sensitivity to change of the Roland-Morris Back Pain Questionnaire: part 2*.* *Phys Ther*, 1998. **78**(11): p. 1197-207.

8. Van der Roer N, Ostelo RW, Bekkering GE, et al. Minimal clinically important change for pain intensity, functional status, and general health status in patients with nonspecific low back pain*. Spine (Phila Pa 1976)*, 2006. **31**(5): p. 578-82.

9. Crosby RD, Kolotkin RL, and Williams GR. Defining clinically meaningful change in health-related quality of life*.* *J Clin Epidemiol*, 2003. **56**(5): p. 395-407.

10. Cohen J. CHAPTER 1 - The Concepts of Power Analysis, in Statistical Power Analysis for the Behavioral Sciences, Cohen J, Editor. 1977, Academic Press. p. 1-17.

11. Fayers PM and Machin D. Quality of life: assessment, analysis and interpretation*.* J*ohn Wiley & Sons*, 2000.

12. Guyatt GH, Bombardier C, and Tugwell PX. Measuring disease-specific quality of life in clinical trials*.* *CMAJ: Canadian Medical Association Journal.* 1986. **134**(8): p. 889-895.

13. Wyrwich KW, Tierney WM, and Wolinsky FD. Further evidence supporting an SEM-based criterion for identifying meaningful intra-individual changes in health-related quality of life*.* *J Clin Epidemiol,* 1999. **52**(9): p. 861-73.

14. Wolinsky FD, Wan GJ, and Tierney WM. Changes in the SF-36 in 12 months in a clinical sample of disadvantaged older adults*.* *Med Care*, 1998. **36**(11): p. 1589-98.

15. McHorney CA and Tarlov AR. Individual-patient monitoring in clinical practice: are available health status surveys adequate? *Qual Life Res*, 1995. **4**(4): p. 293-307.

16. Lydick E and Epstein RS. Interpretation of quality of life changes*.* *Qual Life Res*, 1993. **2**(3): p. 221-6.

17. Beaton DE, Bombardier C, Katz JN, et al. Looking for important change/differences in studies of responsiveness. *J Rheumatol*, 2001. **28**(2): p. 400-5.

18. Dworkin RH, Turk DC, McDermott MP, et al. Interpreting the clinical importance of group differences in chronic pain clinical trials: IMMPACT recommendations*.* *PAIN*, 2009. **146**(3): p. 238-44.

19. Cella D, Bullinger M, Scott C, et al. Group vs individual approaches to understanding the clinical significance of differences or changes in quality of life*.* *Mayo Clin Proc*, 2002. **77**(4): p. 384-92.

20. Guyatt GH. Making sense of quality-of-life data*.* *Med Care*, 2000. **38**(9 Suppl): p. Ii175-9.

21. Testa MA, Interpretation of quality-of-life outcomes: issues that affect magnitude and meaning*.* *Med Care*, 2000. **38**(9 Suppl): p. Ii166-74.

22. U.S. Department of Health Human Services FDA, Center for Drug Evaluation Research. Guidance for industry: patient-reported outcome measures: Use in medical product development to support labeling claims: Draft guidance*.* 2006. **4**: p. 79.

23. Finnerup N B, Attal N, Haroutounian S, et al. Pharmacotherapy for neuropathic pain in adults: systematic review, meta-analysis and updated NeuPSIG recommendations*.* *Lancet Neurol*, 2015. **14**(2): p. 162-73.

24. Duehmke R M, Derry S, Wiffen P J, et al. Tramadol for neuropathic pain in adults*.* *Cochrane Database Syst Rev*, 2017. **6**: p. Cd003726.

25. Schnabel A, Reichl S U, Meyer-Friessem C, et al. Tramadol for postoperative pain treatment in children*.* *Cochrane Database Syst Rev*, 2015(3): p. CD009574.

26. Cepeda M S, Camargo F, Zea C, et al. Tramadol for osteoarthritis: a systematic review and metaanalysis. J Rheumatol, 2007. **34**(3): p. 543-55.

27. Furlan A D, Sandoval J A, Mailis-Gagnon A, et al. Opioids for chronic noncancer pain: a meta-analysis of effectiveness and side effects*.* *CMAJ,* 2006. **174**(11): p. 1589-94.

28. Wiffen P J, Derry S, and Moore R A. Tramadol with or without paracetamol (acetaminophen) for cancer pain*. Cochrane Database Syst Rev*, 2017. **5**: p. CD012508.
